# Submarine groundwater discharge interacts with creek geomorphology to affect eastern oyster *Crassostrea virginica* growth rates in a coastal Georgia creek

**DOI:** 10.7717/peerj.15837

**Published:** 2023-08-04

**Authors:** John M. Carroll, Walker de la Torre, Jacque L. Kelly

**Affiliations:** 1 Biology, Georgia Southern University, Statesboro, Georgia, United States; 2 Geology and Geography, Georgia Southern University, Statesboro, Georgia, United States

**Keywords:** Eastern oyster, Groundwater, Multiple stressors, Management, Geomorphology

## Abstract

Eastern oysters, *Crassostrea virginica*, are commercially important coastal species that provide many ecosystem services for coastal communities. Unfortunately, 85% of oyster reefs have been lost globally, prompting investments in restoration efforts to rebuild populations. Managers often consider several well-studied environmental and water quality parameters when making restoration site decisions. However, recent research suggests that submarine groundwater discharge (SGD) may play a role in driving the distribution of oysters in some estuaries. Specifically, SGD may result in localized areas of low dissolved oxygen and low pH that could inhibit oyster recruitment and survival. However, SGD may interact with other potential oyster stressors, including creek geomorphology. On point bars, sediment accumulation could alter growth rates of oysters and physiology, and it is possible that the two factors, SGD and creek geomorphology, could interact to impact oyster growth. We conducted a field experiment to examine the effects of SGD and creek geomorphology on oyster growth rates in a marsh-lined tidal creek in Georgia, USA. High and low SGD sites were paired within point bars and cut banks. Oysters were deployed in cages for 72 days and growth rates were determined. We found a significant interaction between SGD and creek geomorphology on oyster growth rates. Oysters grew at significantly faster rates at locations on accretionary point bars regardless of SGD flux, whereas, on erosional cut banks, high SGD flux significantly reduced oyster growth rate relative to low SGD flux. It appears that SGD may negatively influence oyster growth at specific creek locations, likely due to the presence of other stressors. Therefore, it is important to consider potential interacting and confounding stressors when managing oyster populations. As SGD is still a relatively understudied potential stressor for oysters, it is critical to continue to examine how groundwater might influence oysters in other locations and in combination with other stressors. Regardless, this study provides further evidence that SGD should be considered in future management efforts.

## Introduction

Oyster populations have declined by ~85% globally, due to a combination of factors, including overharvest, habitat destruction, and diseases, among others (Beck et al., 2011; Kirby, 2004; Rothschild et al., 1994), leading to ecosystem- and coastal community-level consequences due to lost services, including food provisioning, filtration, shoreline stabilization, and habitat formation, among others (Coen et al., 2007; Pierson & Eggleston, 2014). Because oysters are critically important and have experienced dramatic declines, restoration efforts have targeted creating new oyster reefs to enhance populations and ecosystem services (Coen et al., 2007; Gittman et al., 2016), which requires careful consideration of factors that influence oyster distribution. Restoration efforts consider several parameters for site placement, including important water quality variables, such as salinity and dissolved oxygen (DO), as well as substrate relief, and diseases (Dunn, Eggleston & Lindquist, 2014; Pollack et al., 2012; Starke, Levinton & Doall, 2011). While these parameters may vary from location to location, overall success or failure of restoration efforts are usually attributed to unfavorable abiotic conditions (Baggett et al., 2015). It is likely that understudied and less well-known stressors could also contribute to declines in oyster abundance as well as failed restoration efforts.

An understudied factor which might influence oyster distribution is submarine groundwater discharge (SGD). SGD is often patchy in occurrence, and can be a major input of freshwater and nutrients to estuarine systems (Burnett et al., 2003; Wang et al., 2015), although the impacts of SGD on oysters are still poorly understood. For example, SGD can indirectly increase suspension feeder abundance (Encarnacao et al., 2015) as well as bivalve size and physiological condition (Andriosa et al., 2019), due to increased water column chlorophyll *a* driven by groundwater influenced nutrient loading (Hosono et al., 2012; Hwang et al., 2010). Further, SGD can promote favorable water quality conditions and maintain optimal estuarine salinity, more stable temperatures, and provide necessary nutrients compared to estuaries without groundwater (Spalt, Murgulet & Adbulla, 2020; Wang et al., 2015). Alternatively, SGD could have negative impacts on suspension-feeding bivalves. Contaminants, such as mercury, can be delivered to coastal waters *via* SGD, leading to bioaccumulation in mussels (Laurier et al., 2007). When groundwater re-enters surface waters it could also be a major source of dissolved inorganic carbon (DIC; Wang et al., 2015), altering carbonate chemistry, and can also be very low in DO and high in sulfides (Porbusky et al., 2014). Recent research in Georgia suggests a negative correlation between SGD flux and oyster abundance, likely driven by low pH and low DO groundwater affecting successful oyster recruitment (Carroll et al., 2021).

At our study site, the largest SGD fluxes commonly occur on ‘point bars’ or the accretion bank of a meandering creek, suggesting the impacts SGD has on oysters may be confounded by creek bank geomorphology (Carroll et al., 2021). On meandering tidal creeks, bidirectional currents, ebb-current dominance, and sediment combine to generate point bars and cut banks (Barwis, 1977; Leopold, Wolman & Miller, 1964; Ghinassi et al., 2017). While microscale flow may be altered around the banks (Kasvi et al., 2012), the point bars form by accumulation of sediments along the seaward side of bars (Ghinassi et al., 2017). For suspension-feeding bivalves, an accretionary environment can be problematic-sediment can clog filters and decrease feeding, leading to altered physiological condition over short temporal scales (Bricelj & Malouf, 1984; Ellis et al., 2002). For oysters, partial to complete burial has several negative effects, including interruption of recruitment (Poirier et al., 2021), reduced tissue condition (Colden & Lipcius, 2015), and even burial-induced mortality (Comeau, 2014; Lenihan, 1999; Rose, 1973). Oysters may respond to sediment accumulation and partial burial by increasing shell extension rates (Colden & Lipcius, 2015), which may be a common response in stressful conditions (Galtsoff & Luce, 1930; McCormick-Ray, 2005). In addition, sediment accumulation may interact with other stressors, such as contaminants (Edge et al., 2015) and salinity (La Peyre et al., 2020), and other to negatively affect oysters.

In this study, we sought to examine how SGD and tidal creek geomorphology affected the growth rate of eastern oysters, *Crassostrea virginica*, in Oyster Creek, Georgia, USA. Results from recent research within the creek are complex–overall, there was a negative relationship between SGD and oyster abundance, which may have also been impacted by the geomorphology of the creek (Carroll et al., 2021). Based on previous research, we expected to observe faster growth rates for oysters on point bars where sediment accumulation occurs and burial is possible (Colden & Lipcius, 2015), but we did not expect any effect of SGD on oyster growth rates (Carroll et al., 2021).

## Methods

### Study site

This research was conducted in Oyster Creek, which is located approximately 19 km east of Savanna, Georgia. Oyster Creek is a roughly 3 km long sinuous creek that is lined by *C. virginica* and meanders through extensive intertidal marshes of *Spartina alterniflora* in the Ogeechee River basin. Local marsh sediment is predominately composed of clay followed by lesser amounts of silt and then sand (Alexander, Hodgson & Brandes, 2017). Fringing oyster reefs run parallel to shore and are patchy in distribution. In Georgia, oysters generally recruit from May through October and exhibit rapid growth and maturation rates (O’Beirn et al., 1996). Oyster Creek is an approved area for recreational oyster harvesting from October through May (https://coastalgadnr.org/approvedrecharvestareas).

### Geomorphology of oyster creek

**Figure 1:**
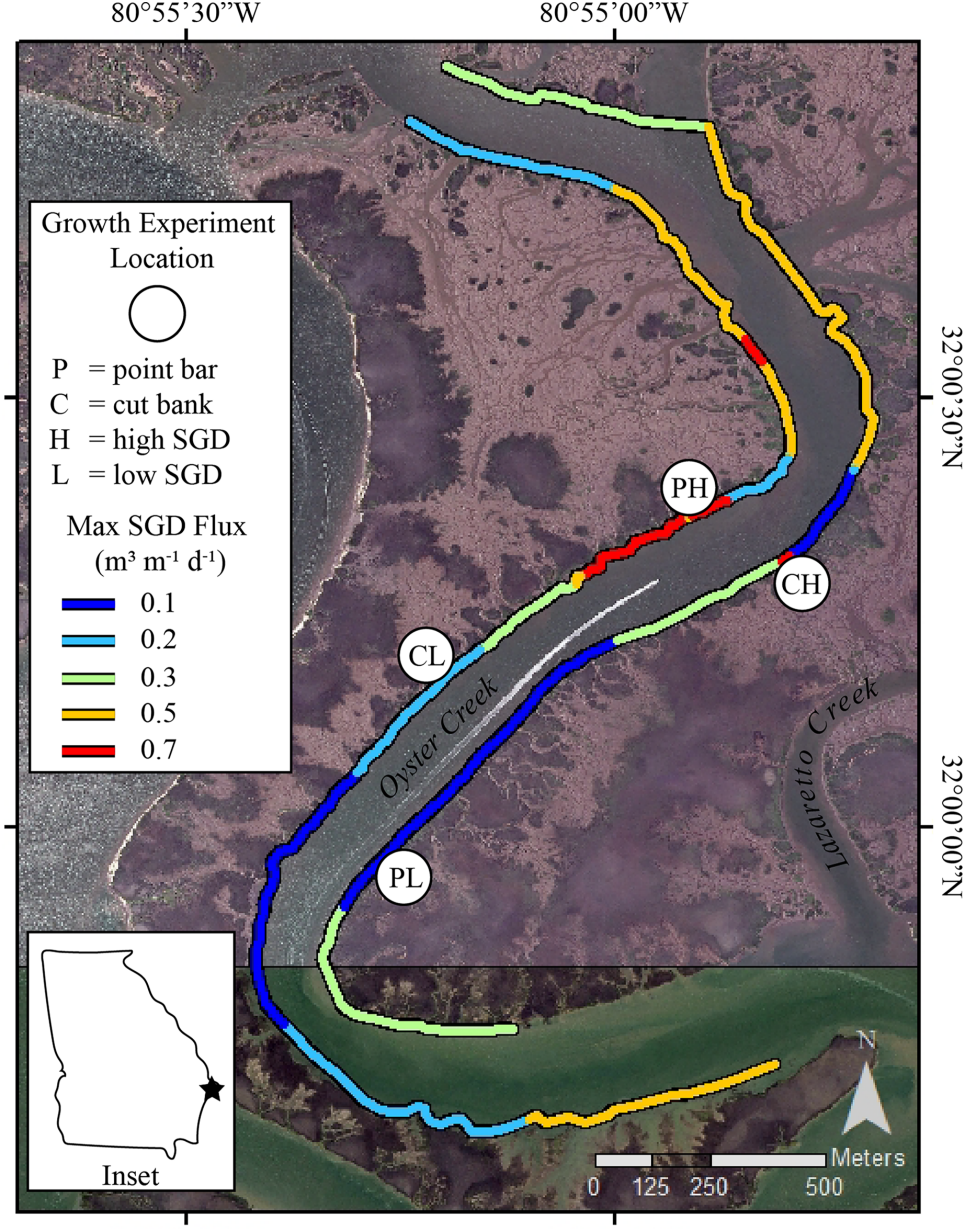
Study sites in Oyster Creek, a tidal creek in coastal Georgia. Growth experiment locations and SGD ûux map of Oyster Creek, GA. Location sites are as follows: PH point bar with high SGD, PL point bar with low SGD, CH cut bank with high SGD, CL cut bank with low SGD. SGD fluxes, measured in 2018, are nonlinearly scaled to maximize display of the range of data. Site labels are directly inland from the study sites. The base map is a 2015 orthoimage from the National Agriculture Imagery Program and has 1 m resolution.

The sinuous nature of Oyster Creek has led to development of point bar and cut bank areas (Fig. 1). Point bars are naturally places of sediment deposition. At these locations, as water flows around a meander, water velocity decreases near the inner bend of the creek thereby dropping sediment grains and creating accretionary banks (Barwis, 1977; Leopold, Wolman & Miller, 1964). Cut banks, by contrast, are erosional banks that develop along the outer bends of the creek where water velocity is greatest (Leopold, Wolman & Miller, 1964). Using geomorphology as a proxy for sediment accumulation and erosion, we selected sites on point bars where sediment accretion occurs and sites on cut banks where sediment erosion occurs for our oyster growth experiments. We recognize that sediment accumulation and erosion rates at point bars and cut banks are site-specific (*e.g*., Coombs et al., 1999). However, measuring sediment accumulation and erosion rates was beyond the scope of this project. Thus, we present the following evidence to support using geomorphology as a proxy for sediment accumulation and erosion at our field site. In Georgia, Jackson (2010) found a relative balance of deposition along point bars and erosion along cut banks with persistent accretion along point bar features in Bear River, which is ~39km to the southwest of our study site. In Lazaretto Creek, which is the creek immediately to the NE of Oyster Creek (Fig. 1), Jackson (2010) calculated a maximum lateral sediment accumulation rate of +0.59 m/year (averaged from 1855 to 2004) on a point bar. Importantly, if point bars are growing laterally, they also must be accumulating sediment vertically (Barwis, 1977; Ghinassi et al., 2017). For cut banks, Coombs et al. (1999) found that the most important parameter that influences erosion rates in neighboring South Carolina is the coverage of oysters and/or shells in the intertidal zone. The cut banks in Oyster Creek have oysters, which may limit overall erosion (Coombs et al., 1999), however, Jackson (2010) calculated a maximum erosion rate of −0.72 m/year (averaged from 1855 to 2004) on a cut bank in Lazaretto Creek (Fig. 1), demonstrating that cut banks near the field site are erosional features. Importantly, creek geomorphological features are easily identified and can be used as a proxy by state management agencies with limited resources to measure and calculate sedimentation rates prior to management decisions.

### Submarine groundwater discharge fluxes

**Figure 2:**
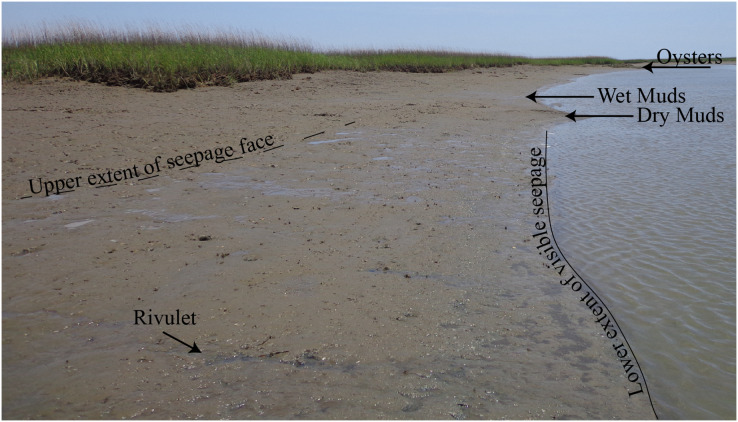
Photo illustrating groundwater flowing out of creekbank. Field site photo showing groundwater actively flowing out of the creekbank between upper extent of the groundwater seepage face (dashed line) and the lower visible extent of the groundwater seepage face (solid line) as well as dry muds where groundwater is not actively flowing out of the creekbank. The photo shows several examples of rivulets of groundwater flow, one of which is labeled as well as an oyster reef.

Surface water surveys for radon-222 (*e.g*., Dulaiova et al., 2010) were conducted at low tide conditions on 25 July 2018 in Oyster Creek. All survey data were used to calculate SGD fluxes (Fig. 1) using a transient mass-balance box model (Dulaiova et al., 2010; Kelly et al., 2018). The exact survey methods and model details are provided in Carroll et al. (2021). The surveys showed variable SGD fluxes that are similar to previously published fluxes for the area (Carroll et al., 2021). Furthermore, the surface water survey from this study showed similar spatial relationships to the 2016 surveys reported in Carroll et al. (2021) indicating that similar groundwater flow paths were active from 2016 through 2018. The sites selected as high SGD during the 2018 surveys were 0.6 L/m/d for the cut bank and 0.5 L/m/d for the point bar, whereas the sites selected as low SGD sites were 0.2 L/m/d for the cut bank and <0.1 L/m/d on the point bar (Fig. 1). Importantly, these are single timepoint measurements, and flux can vary both spatially and seasonally (*e.g*., Carroll et al., 2021). Because flux may vary across season (Michael, Mulligan & Harvey, 2005) and values could change based on model inputs, we also used visual assessments of the presence of groundwater to designate low SGD sites and high SGD sites for the oyster growth rate experiments. Specifically, high SGD sites are characterized by wet mud surface sheen and rivulets (Fig. 2; Carroll et al., 2021), whereas those characteristics are absent at low SGD sites. The visual presence of surface sheens can also be a useful diagnostic tool to identify high SGD sites by state management agencies that do not have radon detectors or other tracers of SGD or the ability to conduct time-intensive coastwide surveys.

Groundwater salinity sampled in Oyster Creek ranges from 28.1 to 32.3 (*n* = 7) across sites, with a mean of 30.1 ± 1.4 (±SD), whereas pH was measured as low as 6.81 and as high as 7.66 (7.34 ± 0.25; mean ± SD)(Carroll et al., 2021). Nutrient ion concentrations (*i.e*., NO_3_^−^, NH_4_^+^, and PO_4_^3−^) were below detection limits of a Dionex Aquion Ion Chromatography System (Carroll et al., 2021). Finally, in previous surveys, there were no relationships between either chlorophyll a concentration (ug/L) or sediment organic content (%LOI) and groundwater flux, while there were strong negative relationships between both pH and DO and groundwater flux (Table 1; Carroll et al., 2021).

**Table 1:** Relationship between SGD and water and sediment variables. Correlations between water and sediment quality metrics based on data from Carroll et al. (2021). Salinity, dissolved oxygen and pH were measured in the water column, while chlorophyll and organic content were from the sediment.

Variable	Correlation coefficient (r)
Salinity	−0.413
DO	−0.580**
pH	−0.771***
Chl a	0.262
Organic content	0.107

**Note:**

Correlation coefficients are shown. Bolded coefficients represented statistically significant correlations,** is 0.05 > α. > 0.01, *** is α. < 0.01.

### Oyster growth rate experiment

We conducted a fully factorial experiment with creek geomorphology (point bar *vs* cut bank) × SGD flux (high *vs* low) field growth experiment by deploying oysters in cages at four locations in Oyster Creek (Fig. 1). Although point bar locations in our study creek typically exhibited the largest SGD fluxes while cut banks exhibit the smallest fluxes (Carroll et al., 2021), there was some spatial variability in flux across geomorphology (Fig. 1). Regardless, the general relationship between creek geomorphology and SGD made it difficult to identify multiple sites for each treatment combination within the creek. Study sites were selected to be on point bars or cut banks. Within the different geomorphology sites, we selected high SGD sites that had visible wet muds and rivulets, and low SGD sites with no visible wet muds (Fig. 2).

A total of 250 oysters were harvested from a local reef within the creek and returned to the lab for initial processing. Oysters were separated and cleaned, assigned a unique ID number, measured for length, and photographed. Groups of five oysters were placed into 8 cm diameter by 20 cm length cylindrical cages made of 6 mm diamond plastic mesh. Cages were fastened to PVC frames and deployed in the field. PVC frames had two levels, one located at the sediment surface and one elevated 10 cm above the sediment. A total of 12 cages and 60 oysters were deployed at each location for a period of 72 days from 11 September to 22 November 2019. Although these dates are at the end of the growing season, growth rates in this study (see results) were comparable to other studies in the southeast that occur during summer months (Dieudonne & Carroll, 2021). At the end of the experimental period, cages were retrieved, oysters were checked for survival, cleaned, and identified. Oysters were then measured and photographed.

Because oysters may exhibit irregular growth patterns, we measured changes in shell area between final and initial oyster photographs using ImageJ (National Institutes of Health, Bethesda, MD, USA) image analysis software. Growth rates may also be affected by initial size, so we calculated daily specific growth rate to standardize growth (Carroll & Finelli, 2014; Kaufmann, 1981) using the following formula:

$Growth = \; \displaystyle{{l{n_{A2}} - \; l{n_{A1}}} \over {{t_2} - {t_1}}}$

where *A*_*2*_ is the area of the oyster shell at the end of the experiment, *A*_*1*_ is the area of the oyster shell at the beginning of the experiment, and *t*_*2*_ *– t*_*1*_ is the time in days for the oyster deployment (Kelly et al., 2011).

### Analysis

Individual growth rates were pooled by cages and cages served as the replicates. We first explored whether cage level (*i.e*., just above the surface *vs* cages placed 10 cm above the sediment surface) impacted growth rates using a t-test. There was no significant difference in growth between cages placed just above the sediment surface *vs* cages placed at 10 cm height (t-test, t = 0.828, *p* = 0.412), so all cages at each site were pooled for further analysis. To examine whether SGD or geomorphology affected oyster growth rates, and to determine whether there was an interaction between the stressors, a two-way ANOVA was conducted using cage means for growth as the response variable and SGD (high *vs* low) and geomorphology (point bar *vs* cut bank) as categorical factors (*n* = 12 per treatment). All pairwise comparisons were made using a Bonferroni Test. Any differences were considered statistically different at an α of 0.05. All analyses were conducted in SigmaPlot.

## Results

During the groundwater survey in July 2018, we collected point measurements for surface water quality at 12 stations along both sides of the well-mixed creek. Temperature ranged from 28.9 to 30.2 °C (29.4 ± 0.3, mean ± SD) while salinity ranged from 29.2 to 30.1 (29.8 ± 0.2). DO was 4.6 ± 0.3 mg/L (4.3 to 5.1) and pH was 7.52 ± 0.05 (7.46 to 7.60). Conditions in the water column adjacent to the four study sites in this study were similar to each other at experiment deployment (Table 2). Except for a lower salinity (27.5 to 28.3), surface water parameters were similar to July 2018 data. SGD was spatially variable throughout the survey, ranging from 0 to 0.7 L/m/d (Fig. 1).

**Table 2:** Water quality metrics of the four study sites. Single time point measurements for temperature, salinity, dissolved oxygen and pH of the four selected study sites, point bar high SGD, point bar low SGD, cut bank high SGD and cut bank low SGD, at the time of cage deployment.

Geomorphology	SGD	Temperature	Salinity	DO	pH
*Point bar*	*High*	31.3	27.5	5.26	7.54
*Point bar*	*Low*	29.7	28.3	4.27	7.49
*Cut bank*	*High*	29.9	27.6	4.34	7.48
*Cut bank*	*Low*	29.8	27.9	4.76	7.52

**Figure 3:**
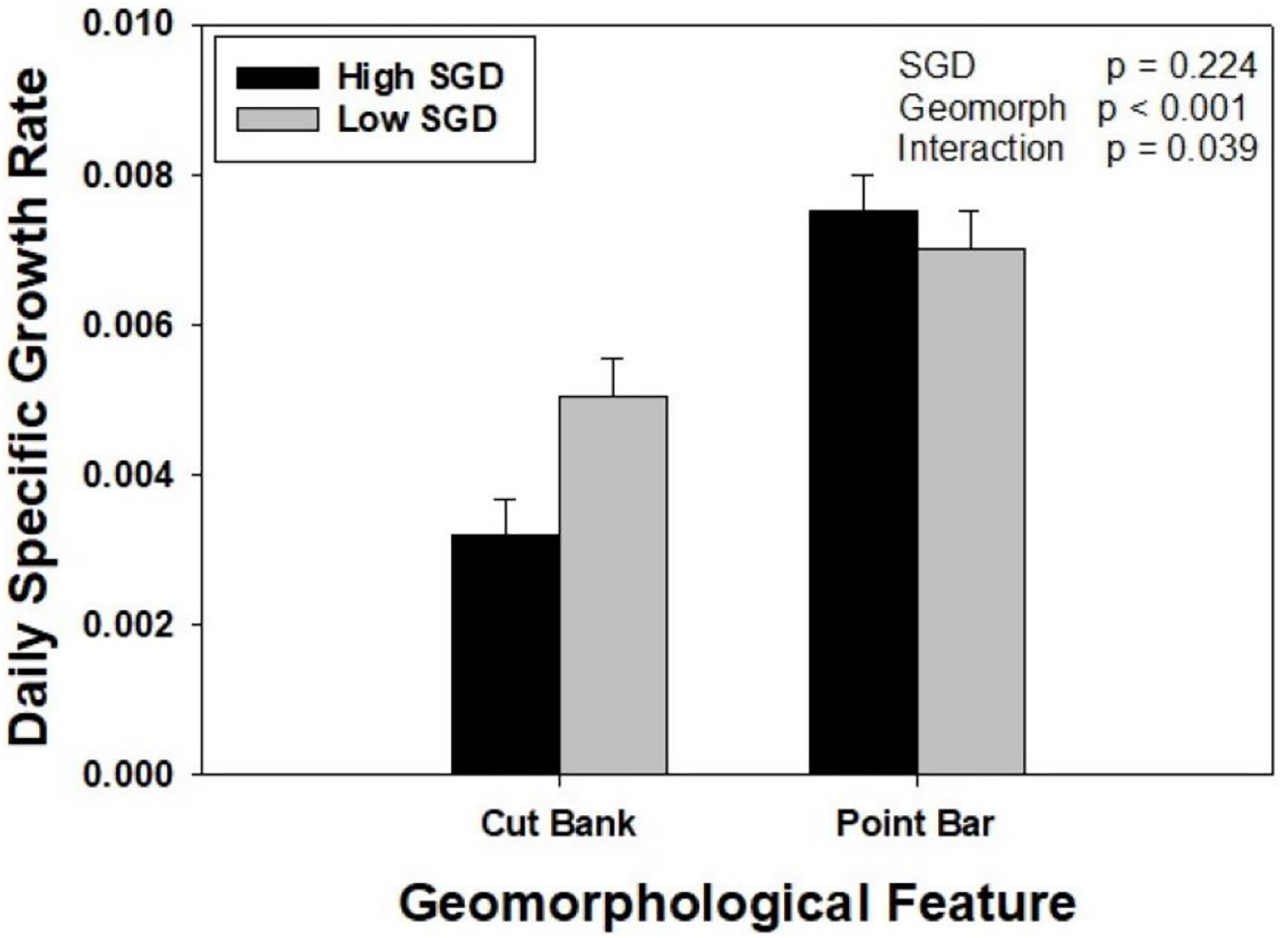
The interaction between sedimentation and groundwater on oyster growth. Oyster daily specific growth rate (growth; ln(cm^2^)d^−1^) at sites of different groundwater flux conditions (high = dark bars, identified by wet muds and rivulets; low = light bars, identified by dry muds) within two different geomorphological features (point bars and cut banks). There was a significant SGD by creek geomorphology interaction (*p* = 0.039). Bars represent means + standard error.

Oysters placed on point bars, regardless of SGD, grew 76% more than oysters placed on cut banks. When considering SGD alone, oysters grew at similar rates. However, there was a significant interaction between groundwater and geomorphology treatments (Two Way ANOVA, F_1,47_ = 31.922, *p* = 0.039). Specifically, within point bar sites, growth did not differ with SGD (*p* = 0.530), whereas oysters at the low SGD cut bank site grew 58% more than oysters at the high SGD cut bank site (*p* = 0.022). Oysters grew twice as fast at the point bar high SGD site compared to the cut bank high SGD site (*p* < 0.001), but only 38% more at the point bar low SGD site compared to cut bank low SGD site (*p* = 0.017; Fig. 3).

## Discussion

Growth rates of intertidal oysters in southeastern US estuaries may be confounded by creek geomorphology that may mask the impacts of other stressors on oyster growth rates in the field. In this study, oyster growth rates were highest on point bars regardless of the presence of SGD. On cut banks, oysters exhibited reduced growth in the presence of relatively high SGD flux compared to the site with low flux. The impacts of groundwater on oysters are still understudied, with some studies suggesting positive impacts (Spalt, Miurgulet & Hu, 2018) and others suggesting negative impacts (Carroll et al., 2021) on populations, likely due to site-specific water quality conditions. This study highlights the importance of exploring potential direct impacts, confounding variables, and designing experiments to disentangle potential interactive effects.

Faster growth rates may be observed in oysters under certain stressful conditions, including burial (Colden & Lipcius, 2015), aerial exposure (Fodrie et al., 2014), and high density (McCormick-Ray, 2005). This study confirms that oysters appear to respond to creek geomorphology by demonstrating faster growth on point bars. Although we did not measure sedimentation directly, it is likely that oysters on point bars are responding to sediment accumulation and potential burial. It is well-established that point bars are sites of high sediment accumulation (Leopold, Wolman & Miller, 1964; Ghinassi et al., 2017), including locally in southeastern estuaries (Barwis, 1977; Jackson, 2010). Oysters have been demonstrated to respond to potential sediment burial by increasing growth rates (Colden & Lipcius, 2015), and it is likely that oysters on point bars in our field study are responding to a similar phenomenon. In an experimental study, Colden & Lipcius (2015) demonstrated that oysters experiencing at least 70% burial exhibited growth rates that exceeded published growth rates in other studies, and the highest growth rates occurred in the 110% burial treatment. In a prior study, we observed burial of oysters at these point bar locations (Carroll et al., 2021; Appendix A). In this study, oysters grew fastest on point bars, regardless of SGD flux, in agreement with studies exploring sediment on oyster growth and morphology (Colden & Lipcius, 2015; Galtsoff & Luce, 1930). Thus, while long-term smothering and burial can be problematic for oyster reefs (Fodrie et al., 2014; Lenihan, 1999), individual oysters compensate for sediment accumulation by increasing growth rates.

The impacts of SGD on oysters are less clear. In some studies, the presence of groundwater introduces necessary nutrients to enhance primary productivity and subsequently secondary productivity (ArrandaCicerol, Herrera-Silbeira & Comin, 2006; Hosono et al., 2012), while also helping to maintain a suitable salinity for oysters (Spalt, Miurgulet & Hu, 2018). However, on cut banks where sediment accumulation does not occur, higher SGD resulted in slower growth of oysters in the present study. Therefore, it does not appear that local oysters are benefiting from additional nutrients or productivity that may occur in areas of high SGD flux (Hosono et al., 2012). Within the intertidal distribution of oysters throughout the southeast US, oyster density and biomass peaks in SC and GA (Byers et al., 2015), where growth rates are high (Dieudonne & Carroll, 2021; Harding, 2020; Manley et al., 2010), suggesting that local oysters are not likely food-limited (O’Beirn et al., 1996). Further, there was no evidence of increased nutrients or chlorophyll associated with high groundwater fluxes in earlier studies at our experimental sites (Carroll et al., 2021). Therefore, it does not appear that the presence of groundwater is yielding higher productivity at our study sites.

In Georgia, high groundwater flux has a strong, negative relationship with oyster density, which was attributed to recruitment interruption by poor water quality in the microlayer surrounding oysters and structure in high groundwater flux areas (Carroll et al., 2021). While we did not measure groundwater quality in this study, previous studies in the region have demonstrated that groundwater can exhibit severe hypoxia to anoxia, have low pH, and potentially high sulfide concentrations (Carroll et al., 2021; Porbusky et al., 2014), all of which contribute to low oyster growth (Gobler et al., 2014; Walbusser, 2011) and reduce larval survival and subsequent recruitment (Miller et al., 2009; Talmage & Gobler, 2010). Poor water quality conditions likely contributed to the lower overall growth rates exhibited at the high SGD cut bank site, as low DO and low pH can reduce post-set and juvenile oyster growth (Baker & Mann, 1992; Talmage & Gobler, 2011). We unfortunately did not measure water quality in the microlayer surrounding our caged oysters, but prior research suggests strong negative correlations between SGD and both DO and pH in this creek (Carroll et al., 2021). Furthermore, the similar spatial patterns of groundwater discharge from the 2016 data reported in Carroll et al. (2021) and the data presented here suggests that the groundwater outflow sites in the creek remain active over time scales of at least years and can be considered a chronic, long-term stressor on oysters. Overall, these studies provide evidence that SGD appears to be having a negative impact on intertidal oyster populations in Georgia.

In an earlier study at the same study sites, oyster length increased with SGD, when present, and oysters exhibited relatively fast and similar growth rates across a range of SGD in a caged growth experiment (Carroll et al., 2021). This led the authors to conclude that if established, oysters may survive and grow in areas of SGD (Carroll et al., 2021), as has been demonstrated in other studies where groundwater has been shown to be beneficial to oysters (Spalt, Murgulet & Adbulla, 2020). However, the surveys of population density in Oyster Creek suggested an overall negative impact of SGD on oysters locally (Carroll et al., 2021). It is possible that the earlier findings regarding growth rates in Georgia were due to interactions between SGD and geomorphology within the creek. In the original study, SGD tracked the geomorphology of the creek bank (*i.e*., Fig. 1), with higher fluxes occurring on point bars where accretion also occurs. In that study, since the interest was in SGD, sites were selected where SGD was expected to be highest, and those sites happened to be on point bars where oyster burial did occur (Appendix A). Given the responses of oysters to sediment burial (*i.e*., Colden & Lipcius, 2015), previous findings of high growth in high SGD areas in Georgia estuaries could have been confounded by creek geomorphology and associated sediment accumulation. The current study provides additional evidence that SGD negatively impacts oysters in Georgia and demonstrates the need to consider experimental artifacts that might be caused by site selection and confounding variables in field studies.

Although oysters create hard-substrate reefs, they are often surrounded by soft, muddy sediments, where lower portions of reefs may get buried (Fodrie et al., 2014; Lenihan, 1999). In Georgia, oysters in muddy habitats tend to be more elongated, likely as a response to sediment accumulations (Galtsoff & Luce, 1930). These conditions are likely contributing to the complex responses of oysters to SGD flux in this region. In the field, the relationship between SGD flux and suspension feeders like oysters appears context-dependent, and influenced by local conditions. In Georgia, areas of high relative flux have been linked to low oyster density and reduced recruitment (Carroll et al., 2021), and now, reduced growth. These effects also appear to be interacting with creek geomorphology, and the accumulation of muddy sediments on point bars (Jackson, 2010) may mask individual oyster responses. Regardless, this research provides important information for managers when making decisions about restoration site-selection. Importantly, the results suggest that managers can potentially use creek geomorphology and visible groundwater cues to make site decisions, although additional surveys should be conducted in multiple creeks.

In conclusion, the current study highlights the importance of considering potentially confounding variables that may interact to impact oysters, especially in field studies. Multiple stressors often interact with each other to affect individual responses (Cole et al., 2016; Lenihan et al., 1999), and can ultimately have cascading effects to the ecosystem level (Hewitt, Ellis & Thrush, 2016; Rogers-Bennett & Catton, 2019). While the negative impacts of sedimentation on oyster reefs and restoration projects are well known (Caretti, Bohnenstiehl & Eggleston, 2021; Colden, Latour & Lipcius, 2017; Fodrie et al., 2014), the effects of groundwater are still poorly defined and highly complex. Research should continue to explore the site-specific responses to multiple stressors, since in other regions, SGD may be beneficial for oysters (Hosono et al., 2012; Spalt, Murgulet & Adbulla, 2020) and may not lead to similar outcomes. Additionally, in creeks that have less sinuous geomorphology (*i.e*., those lacking in point bars and cut banks), sediment accumulation and other stressors like SGD may not interact, suggesting future study to fully understand the relationships between oysters and potential geological stressors. This research demonstrates the need to consider previously understudied factors, such as SGD, that could negatively impact oyster demographics as well as to consider how multiple stressors may interact to affect oyster restoration and management.

## Supplemental Information

10.7717/peerj.15837/supp-1 Supplemental Information 1 Burial of oyster growth cages on a point bar. Prior growth experiments for the Carroll et al., 2021 study were conducted on point bars where SGD was also highest. Many of the oyster cages experienced burial, as shown, on these point bar sites, further illustrating sediment accumulation does happen at point bars in our study area.

10.7717/peerj.15837/supp-2 Supplemental Information 2 Oyster daily specific growth rate data. Daily specific growth rate data for caged oysters placed at sites with different sedimentation conditions (high and low) and submarine groundwater discharge conditions (high and low). “Metadata” sheet contains metadata, and “Data” sheet contains the cage mean growth rates across the different treatments.

## Additional Information and Declarations

### Data Availability

The following information was supplied regarding data availability: The raw data used for analysis of oyster growth rates are available in the Supplemental File.
