# Supplementary figures and images for: Submarine groundwater discharge interacts with creek geomorphology to affect eastern oyster Crassostrea virginica growth rates in a coastal Georgia creek
Source: PeerJ. 2023 Aug 4;11:e15837. doi: 10.7717/peerj.15837 (PMC10405797; doi:10.7717/peerj.15837)

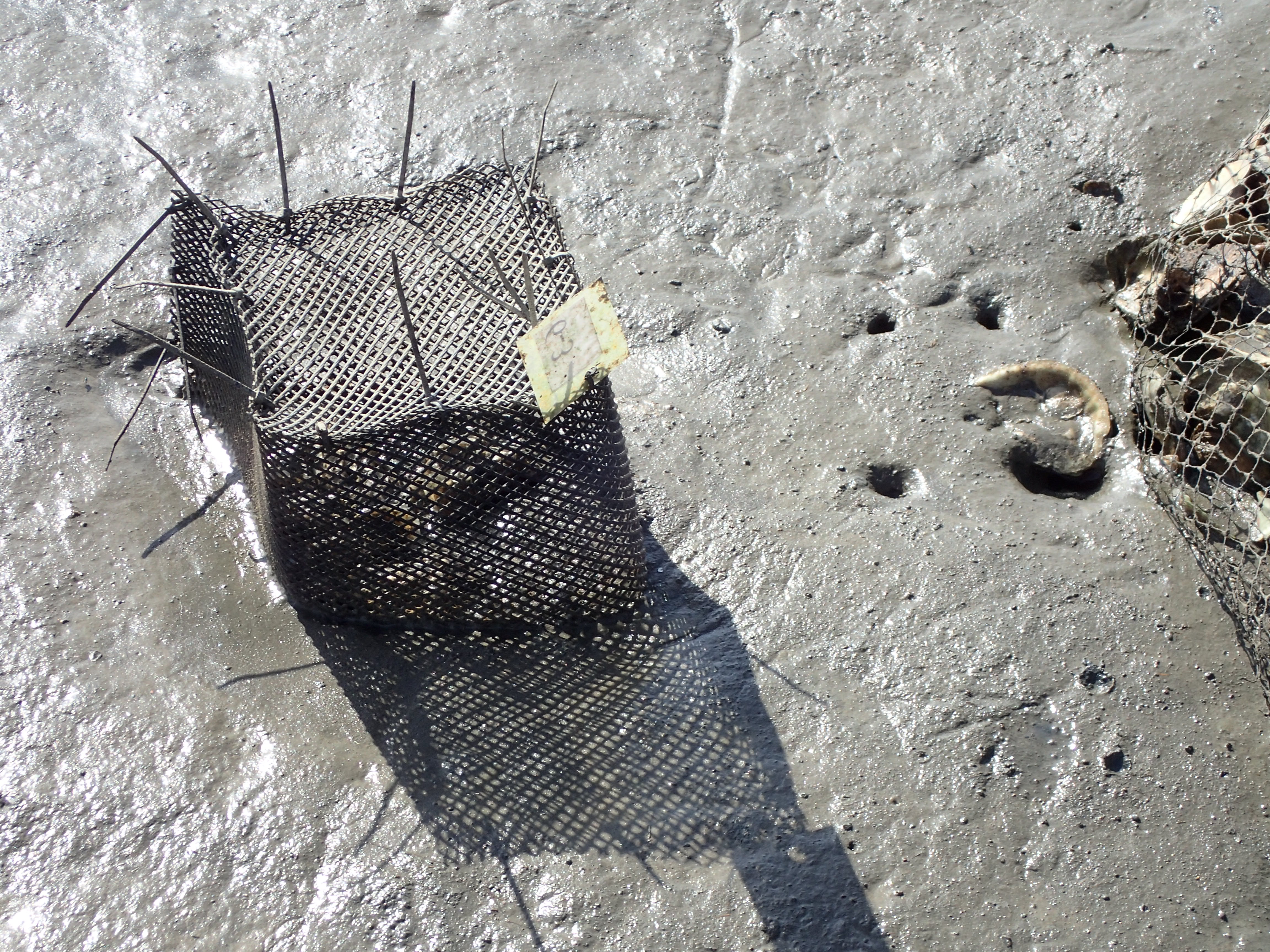

Supplement: Supplemental Information 1 — Prior growth experiments for the Carroll et al., 2021 study were conducted on point bars where SGD was also highest. Many of the oyster cages experienced burial, as shown, on these point bar sites, further illustrating sediment accumulation does happen at point bars in our study area. [file peerj-11-15837-s001.jpg]
